# Effects of rasagiline combined with levodopa and benserazide hydrochloride on motor function and homocysteine and IGF-1 levels in elderly patients with Parkinson’s disease

**DOI:** 10.1186/s12883-023-03411-3

**Published:** 2023-10-06

**Authors:** Yifan Yang, Feng Gao, Li Gao, Jiaodan Miao

**Affiliations:** 1 Department of Neurology, Affiliated Hospital of Southwest Jiaotong University & Chengdu Third People’s Hospital, Chengdu, Sichuan 610000 China; 2 Southwest Jiaotong University, Chengdu, Sichuan 610000 China

**Keywords:** Parkinson’ s disease, Levodopa and benserazide hydrochloride, Rasagiline, Hcy, IGF-1, Motor function

## Abstract

**Background:**

During the course of their illness, people with Parkinson’s disease may see changes in their insulin-like growth factor (IGF-1) and serum homocysteine (Hcy) indices. In this study, patients with intermediate to severe Parkinson’s disease were examined for how Resagiline and levodopa and benserazide hydrochloride affected their motor performance, serum levels of homocysteine (Hcy), and insulin-like growth factor (IGF-1).

**Methods:**

From June 2020 to December 2021, a total of 100+ cases of Parkinson’s patients over 60 years old in the middle and late stages of Parkinson’s were seen in the outpatient and inpatient departments of the Third People’s Hospital of Chengdu City and had a detailed observation record, and according to the inclusion criteria, the patients who met the criteria were randomly grouped into a clinical observation group and a control group. The subjects in the control group received only levodopa and benserazide hydrochloride treatment, while the observation group was treated with Resagiline in combination with the clinical control group. The total treatment observation period was 1 year for both groups, and the motor function and serum Hcy and IGF-1 indexes of both groups were compared after the end of treatment.

**Results:**

We randomly and evenly grouped 64 patients who met the requirements of the inclusion criteria into a clinical observation group and a control group, each with 32 patients, from among 168 patients over 60 years of age with detailed observation records in the middle and late stages of Parkinson’s. After the 1-year observation period, we found that the total effective rate after treatment in the clinical observation group (93.75%) and significantly higher than that in the control group (68.75%) (*P* < 0.05); after 1 year of treatment, the UPDRS score decreased in both groups, and the observation group was significantly lower than the control group (*P* < 0.05); after treatment, serum Hcy decreased and IGF-1 increased in both groups, and the observation group was higher than the control group mean values (*P* < 0.05).

**Conclusions:**

In patients with Parkinson’s disease who are in the middle and late stages of the disease, the administration of Resagiline combined with levodopa and benserazide hydrochloride can significantly lower the body’s serum Hcy level, significantly raise IGF-1 levels, and significantly improve motor function in patients with Parkinson’s disease. It can also have significant therapeutic effects.

## Background

Parkinson’s disease (PD), a neurodegenerative condition that can have an impact on various bodily systems, is brought on by an abnormal decline in striatal dopamine levels [1]. The World Health Organization estimates that there are presently 6 million people worldwide who have PD, with China accounting for over half of all cases. A projected 9 million people worldwide will have Parkinson’s disease (PD) by 2030 as a result of the rapidly aging global population, with more than 5 million of these patients being Chinese [2]. A significant drop in nigrostriatal dopamine levels is a hallmark of Parkinson’s disease (PD), a neurodegenerative condition that progresses over time and is associated with a number of motor symptoms like bradykinesia, resting tremor, and dystonia as well as postural gait abnormalities and other non-motor symptoms [3]. Additionally, it has been noted that some Parkinson’s disease (PD) non-motor symptoms, such as neuropsychiatric symptoms, sleep disorders, postural hypotension, autonomic dysfunction, and sensory dysfunction, manifest before motor symptoms. During this period, which may last 5 to 20 years, the patients’ quality of daily life will likely be significantly impacted.

Age of onset is a key factor in neurodegenerative illnesses because it is connected to different phenotypes and disease progression [4]. As a result of the examination of more PARK genes [5], more PARK gene variations were discovered in people with early-onset Parkinson’s disease (EOPD), which also demonstrated that certain symptoms are connected to unusual PARK gene variants [6]. According to research, homocysteine, immunoinflammatory processes, excitatory neurotoxicity, oxidative stress damage, apoptosis, and autophagy all have a role in the etiology of Parkinson’s disease [7]. Experimental research has demonstrated that Hcy can be neurotoxic and excitatory to the substantia nigra among them. Dyskinesia, a sign of potential neurodegeneration brought on by a change in the equilibrium of striatal activity, may be linked to Hcy [8, 9]. It’s possible that Hcy’s toxic effects are what cause dyskinesia and motor fluctuations [10]. Impaired cognitive and motor dysfunction may also result from high Hcy concentrations in PD patients. Also, investigated in this initiative is the neurotrophic factor insulin-like growth factor 1 (IGF-1), which is crucial for the survival of neurons and brain function. In idiopathic Parkinson’s disease, IGF-1 resistance—characterized by elevated levels of circulating IGF-1 and diminished IGF-1 function—is linked to disease progression and cognitive decline [11–14].

A series of common complications and somato-psychological adverse reactions will occur in the most advanced PD patients who have not undergone formal clinical examination and evaluation and formal treatment, as the disease progresses and worsens, severely impairing their ability to perform daily living activities and even gradually developing to complete loss of the most basic autonomous living abilities [15].

PD is an incurable disease that progresses continuously over time, so early diagnosis and early treatment are extremely crucial to improve the prognosis of Parkinson’s patients. A large number of clinical trials provide evidence that health-related quality of life can be greatly improved through early diagnosis and establishment of motor and other physical measures, appropriate timing of dopaminergic treatment, and strategies to delay and treat levodopa-related motor complications and symptoms associated with non-motor Parkinson’s disease [16]. Among them, pharmacotherapies are the main treatments, and currently the main drugs used include: levodopa and benserazide hydrochloride, monoamine oxidase (MAO) inhibitors, catechol oxygen methyltransferase (COMT) inhibitors, dopamine agonists (Das) (e.g., piribedil is a non-ergot selective Dopamine agonists (e.g., piribedil is a non-ergot selective dopamine D2 and D3 receptor agonist; pramipexole is a non-ergot D2/D3 receptor agonist; ropinirole is a non-ergot selective dopamine D2 receptor agonist; rotigotine produces anti-PD effects through continuous stimulation of D3/D2/D1 receptors; apomorphine is a potent D1 and D2 receptor agonist), dopamine-releasing drugs (e.g., amantadine), and anticholinergic agents (e.g., benzhexol).PD is treated clinically with the levodopa and benserazide hydrochloride tablets. It can effectively control the rapid progression of PD through direct supplementation of exogenous dopamine, but due to long-term continuous use, not only will the effect of drug therapy be greatly reduced, but also will significantly aggravate the motor dysfunction. According to reports [17] in the literature, levodopa medication therapy may also have an impact on plasma Hcy levels in PD. According to a research by Dorszewska and Kozubski [12], long-term levodopa medication may cause high Hcy levels in individuals.

There are two isoforms of the enzyme monoamine oxidase (MAO), which breaks down monoamines: MAO-A and MAO-B. The MAO-B inhibitor (MAO-BI) slows down the breakdown of dopamine and the activity of type B monoamine oxidase, which may have neuroprotective effects [18]. It also delays the worsening of symptoms. Resagiline is a strong, highly bioselective, irreversible second-generation MAO-B inhibitor that inhibits MAO-B five to ten times more than first-generation sellegrin without being affected by dietary intake [19, 20]. Additionally, it enhances dopamine secretion by inhibiting dopamine breakdown in patients and increasing dopamine buildup. Rasagiline can be used as a combination therapy with the levodopa and is often chosen for patients with significant comorbidities in the middle and late stages of PD [21]. It can also be used as a single agent for the first treatment of newly diagnosed early PD patients [22]. In order to examine and investigate the improvement in motor function and the impact of resagiline and levodopa and benserazide hydrochloride on serum Hcy and IGF-1 levels in patients with intermediate and advanced PD, this study will adopt a retrospective methodology.

## Methods

### Clinical data

One hundred sixty-eight Parkinson’s patients who received care at the Third People’s Hospital of Chengdu’s outpatient and inpatient units between June 2019 and June 2021 were chosen, and 64 of them who met the criteria for inclusion were randomly split into the observation group and the control group, with 32 patients in each group. 18 male and 14 female patients made up the observation group. Their ages ranged from 65 to 75 years, with a mean of 69.38 ± 5.74 years. The length of their illnesses ranged from 5.8 to 10.8 years, with a mean of 7.20 ± 1.10 years. 19 male and 13 female patients made up the control group. Their ages ranged from 64 to 76 years, with a mean age of (68.58 ± 5.68) years. The length of their illnesses ranged from 6.1 to 9.8 years, with a mean length of (7.8 ± 1.30) years. In terms of gender, age, and length of disease, there was no statistically significant difference (*P* > 0.05) between the two groups, and the differences were equivalent. Patients willingly agreed to participate in this study and signed an informed consent form. This study was ethically approved by the Third People’s Hospital of Chengdu with approval number: 2020-T-36.

### Inclusion and exclusion criteria

#### Inclusion criteria

① Patients were aged > 60 years old; ② Complied with the diagnostic criteria for Parkinson’s disease in Chinese Parkinson’s Disease Diagnostic Criteria (2016 edition) (they are the same as MDS Criteria); ③ All enrolled patients had Hoehn-Yahr grading of grade 3–4 and were in the middle and late stages of Parkinson’s disease with end-dose fluctuations. Pre-enrolled patients with moderate to advanced Parkinson’s had only been treated with dobasazide tablets. ④ No contraindications to the drug; ⑤ Good compliance, able to accept 1 year of treatment.

#### Exclusion criteria

① Patients aged ≤ 60 years; ② Patients with a history of drug allergy; ③ Patients with motor dysfunction or cognitive dysfunction caused by other combined lesions; ④ Patients with other combined physical diseases of severe severity such as a malignant tumor.

### Treatment

The control group received only levodopa and benserazide hydrochloride tablets: oral, 0.125 g/dose for the first dose, 3 times/d. After 1 week of continuous dosing, the dose was changed to 0.125 g 2. Per week (depending on the patient’s condition), and the maximum dose should not exceed 1.5 g in 1 day. The observation group was given the combination treatment of Resagiline + levodopa and benserazide hydrochloride tablets, in which the dose and regimen of levodopa and benserazide hydrochloride tablets were the same as above, and Resagiline was administered orally at 1 mg once daily. The duration of treatment for both groups was 1 year. In addition, patients in both groups were given the same routine rehabilitation training.

### Observation indexes

To compare the differences in the levels of the relevant serum biochemical indices Hcy and IGF-1 as well as the clinical effectiveness of the two groups after a year of treatment. First, the patients’ fasting venous blood was drawn, the serum was separated, and the levels of serum Hcy and IGF-1 were assessed using an enzyme-linked immunosorbent assay. The H-Y grading system, which is divided into 0–5 levels from mild to severe, with higher levels indicating more severe impairment of specific motor functions, can efficiently and appropriately be used to measure the severity of motor impairment and its influence on everyday living. The patients’ motor function, daily activities, emotions, and complications were assessed using Parkinson’s Disease Rating Scale (UPDRS), which yielded a final score of 44 points.

### Efficacy assessment indexes

Comprehensive clinical efficacy: apparent efficacy means that patients have significant clinical efficacy and improvement in muscle tone and motor function, and can carry out general daily life activities; effective is mainly manifested as patients’ muscle tone and motor function have been improved accordingly, and can assist in daily life activities; ineffective is defined as patients’ clinical symptoms have not improved or even further aggravated after treatment with drugs. Total effective rate = apparent rate + effective rate. According to the level of UPDRS score reduction after treatment, effective: UPDRS score reduction of ≥ 50%; effective: UPDRS score reduction of 20 ~ 50%; ineffective: UPDRS score reduction of < 20%. Total effective rate = (number of effective cases + number of effective cases) / total number of cases × 100%.

### Data analysis

SPSS 21.0 software was used for statistical analysis. χ2 test was used for count data (%) and t-test was used for measurement data (± s), and statistical differences were found at *P* < 0.05.

## Results

### Comparison of clinical efficacy between the two groups of patients’ treatment

After 1 year of treatment, the total effective rate of patients in the observation group was significantly higher than that in the control group, and the difference was statistically significant (*P* < 0. 05), see Table 1.

**Table 1:** Comparison of clinical efficacy between patients in the observation group and the control group (cases (%))

Group	Significant effect	Effective	Ineffective	Total effective
Observation group (32 cases)	12 (37.5%)	18 (56.25%)	2 (6.25%)	30 (93.75%)
Control group (32 cases)	8 (25%)	14 (43.75%)	10 (31.25%)	22 (68.75%)

### Comparison of H-Y grade and UPDRS score between the two groups before and after treatment

The change of patients’ condition can be assessed from motor function, daily activities, mood, cognition, and complications according to UPDRS score, see Table 2. After treatment, the H-Y score and UPDRS score of patients in both groups decreased significantly compared with those before treatment, and patients in the observation group were significantly lower than those in the control group. After treatment, the differences were statistically significant (*P* < 0. 05), see Table 3. The patients’ H-Y scores and UPDRS scores were better than before, indicating that the patients’ clinical symptoms such as motor function, daily activities, mood, cognition, and complications were improved. In this study, we found that this part of UPDRS III had the greatest change, and the UPDRS III scores of the patients in both groups were reduced after treatment compared with before treatment, and the UPDRS III scores of the observation group were lower than those of the control group, and the differences were all statistically significant (all *P* < 0.05). The differences were statistically significant (all *P* < 0.05), see Table 4.

**Table 2:** Comparison of changes in disease characteristics before and after treatment between the two groups according to UPDRS scores

	Observation group		Control group	
	Before Treatment (score)	After Treatment (score)	Before Treatment (score)	After Treatment (score)
Spiritual, behavioral and emotional	6.13 ± 1.12	3.28 ± 0.66	5.84 ± 0.32	4.28 ± 0.88
Daily Activities	16.72 ± 1.06	10.32 ± 0.54	17.21 ± 0.68	14.32 ± 0.96
Motion function	12.46 ± 0.8	7.86 ± 0.82	11.08 ± 1.01	9.77 ± 0.46
Post-treatment complications	3.38 ± 0.56	1.19 ± 0.34	4.01 ± 0.97	2.27 ± 0.65
Rating	38.69 ± 3.54	22.56 ± 2.36	38.14 ± 2.98	30.64 ± 2.95

**Table 3:** Comparison of H-Y scores and UPDRS scores before and after treatment in the two groups

	H-Y grading (grade)		UPDRS score (points)	
	Before Treatment	After treatment	Before Treatment	After treatment
Observation group (32 cases)	3.8 ± 0.5	2.0 ± 0.4	38.69 ± 3.54	22.56 ± 2.36
Control group (32 cases)	3.7 ± 0.4	2.3 ± 0.6	38.14 ± 2.98	30.64 ± 2.95
t	0.883	-2.353	0.672	-12.099
*P*	0.268	0.027	0.316	< 0.001

**Table 4:** Comparison of UPDRS III scores between the two groups of patients before and after treatment (score, ± s)

	UPDRSIII	
	Before Treatment	After treatment
Observation group (32 cases)	30.41 ± 5.75	19.41 ± 4.20
Control group (32 cases)	30.21 ± 5.52	23.44 ± 3.19
t	0.142	4.322
*P*	0.888	0.000

### Comparison of serum Hcy and IGF-1 levels before and after treatment in the two groups

Following 1 year of treatment, we saw that the serum Hcy levels in the two gatherings were altogether lower and IGF-1 levels were essentially higher than before treatment, and the serum Hcy and IGF-1 levels in the perception bunch were fundamentally better compared to those in the benchmark group, and the distinctions were measurably huge (*P* < 0. 05), see Table 5.

**Table 5:** Comparison of serum Hcy and IGF-1 levels before and after treatment in two groups ($\overline{x}\pm \mathrm{s }$)

	Hcy (μmmol/L)		IGF-1(nmmol/L)	
	Before Treatment	After treatment	Before Treatment	After treatment
Observation group(32cases)	16.35 ± 2.68	10.35 ± 1.68	8.6 ± 2.2	11.4 ± 1.6
Control group (32 cases)	16.95 ± 2.88	12.34 ± 1.72	8.2 ± 2.8	9.5 ± 1.2
t	0.575	-4.682	0.635	5.374
*P*	0.336	< 0.001	0.324	< 0.001

## Discussion

PD is quite possibly of the most well-known neurological problem in the old. The typical period of beginning is 55 years, and the gamble of creating PD increments fivefold by age 70 [23]. There are two types of PD, irregular (SPD), which influences 95% of all patients, and familial (FPD), which represents around 5–10% of all causes. The etiology of SPD is believed to be obscure, while FPD is related with hereditary transformations at the Recreation area locus. Elevated degrees of Hcy in PD might build the gamble of this neurodegenerative illness through harmful impacts on dopaminergic neurons. In vitro examinations performed on human dopaminergic neurons have shown a huge expansion in neurotoxicity related with high Hcy levels [24]. High Hcy fixations in patients with PD may likewise prompt impeded mental and coordinated abilities and depression [25]. Reports [26] in the writing propose that plasma Hcy levels in PD are likewise impacted by levodopa drug treatment. Dorszewska and Kozubski’s [12] study showed that the patients generally defenseless against Hcy neurotoxicity seemed, by all accounts, to be the individuals who got levodopa treatment during the initial 5 years.

It was found that the use of MAO-B inhibitors may also provide sufficient benefit when patients experience mild motor symptoms, but with a lower risk of adverse events [27]. In addition to being more convenient for patients, Resagiline as an adjuvant to levodopa can greatly minimize the duration of the “off state” and ameliorate the symptoms of motor fluctuations brought on by levodopa treatment [21]. Most of patients will be combined with engine vacillation jumble after over 4 years of levodopa treatment, and most of the patients will be joined with swaying issue assuming they have taken the medicine for over 10 years, as indicated by long periods of clinical contextual analyses. Levodopa can work on the clinical side effects of Parkinson’s, yet it has restricted viability in side effect control alone, and as the portion of levodopa expands, it won’t just goal a comparing expansion in the frequency of engine complexities, yet in addition produce various levels of cardiovascular framework, urinary framework, gastrointestinal and other unfavorable impacts [28]. The inhibition of MAO-B by Resagiline also increases the availability of phenylethylamine, which enhances striatal dopamine release [29] and protects damaged neurons and greatly reduces the long-term dose of levodopa drugs, which not only significantly reduces the incidence of adverse effects, but also fully ensures the effectiveness of treatment. Resagiline tablets can be used both directly in the monotherapy of Parkinson’s disease and as an adjuvant to levodopa drug therapy in Parkinson’s patients with severe motor fluctuation complications, and its clinical treatment effect is improved significantly [30].

The latest results of this study showed that through about 1 year of combined drug and rehabilitation therapy, the H-Y classification and UPDRS scores of the patients in the observation group were significantly lower than those of the control group, and the UPDRS III scores of the patients in the observation group were all significantly lower than those of the control group. This suggests that relative to levodopa therapy alone, the addition of rasagiline adjuvant therapy to levodopa is more effective in improving the motor function of patients with Parkinson’s disease, reducing the incidence of end-of-agent fluctuations of motor complications, and leading to a significant improvement in the motor function of patients with Parkinson’s disease. Homocysteine (Hcy) is an intermediate product of amino acid metabolism, which can lead to a decrease in the number of dopaminergic neurons, increase dopaminergic neuron damage [31], and damage the substantia nigra, resulting in a series of dyskinesia symptoms.

The results of this analysis showed that after 1 year of treatment, the degree of hcy reduction in patients in the observation group was significantly better than that in the control group, and the differences were all statistically significant (*P* < 0. 05), which may be explained by the neuronal protective function of Resagiline, a drug that also prevents dopamine neurons from Hcy-mediated oxidative stress damage. In addition, IGF-1, whose main chemical molecular structure and function are similar to insulin, is involved in cellular energy metabolism and growth catabolism, and is a major central neuromodulatory factor in the CNS. IGF-1 can initiate intracellular signaling pathways and regulate intracellular nutrient metabolism, thereby regulating cell proliferation, differentiation, and other processes, as well as reducing oxidative damage, decreasing neurotoxic It can also reduce oxidative damage, reduce the production of neurotoxic substances and inhibit apoptosis, thus playing a neuronal protective role [32]. The results of this study showed that the serum IGF-1 level in the observation group was significantly higher than that before treatment, and the difference was statistically significant (*P* < 0. 05), which may be due to the fact that the combination of Resagiline can inhibit the regulatory function of monoamine oxidase B, thus enhancing the release of dopamine in the body and blocking the uptake of dopamine, which plays a role in protecting dopaminergic neurons.

In summary, Resagiline, as a second-generation B-type monoamine oxidase (MAO-B) inhibitor, improves brain dopamine levels by inhibiting MAO-B activity, thereby increasing dopaminergic content and prolonging the maintenance effect of exogenous dopaminergic drugs, thereby improving the effect of combination therapy and post-drug administration and controlling the development of remission [33]. The combination of levodopa and benserazide hydrochloride can also significantly reduce the patient’s serum Hcy concentration level and enhance serum IGF-1 concentration, thus improving the patient’s motor function, reducing the occurrence of adverse reactions, and alleviating the patient’s end-of-dose fluctuations with significant effects [34]. It is worthy of our clinical promotion and clinical application. Nonetheless, in light of the fact that the aggregate sum of clinical information in this undertaking is moderately little, and further nitty gritty and extensive clinical and ceaseless follow-up is required, the aggregate sum of clinical information and the profundity of constant subsequent should be additionally expanded in this review, in order to explain the drawn out clinical symptomatic targets and impacts in more profundity.

## Data Availability

All data generated or analysed during this study are included in this published article.
